# PGViS: Personal Genome Variant interpretation Score for lung cancer genomes

**DOI:** 10.64898/2026.08.01.742250

**Published:** 2026-08-06

**Authors:** Pallavi Surana, Pratik Dutta, Paolo Boffetta, Ramana Davuluri

## Abstract

Inherited lung cancer risk arises from both protein-coding and non-coding germline variants, but the functional non-coding component is largely uncharacterized. Genome-wide association studies and polygenic risk scores identify tag variants, not causal ones. Neither resolves which regulatory element is perturbed. DNA foundation models such as DNABERT decode non-coding variant effects directly from sequence, without a large GWAS cohort. What is missing is a patient-level framework linking these predictions to population-level variant prevalence. We present PGViS (Personal Genome Variant interpretation Score), a statistical framework that quantifies individual non-coding germline regulatory risk in non–small cell lung cancer (NSCLC). PGViS integrates three variant-level signals: DNABERT-predicted disruption at transcription factor binding and splice sites, the cancer v/s reference alternate allele frequency shift, and a regulatory interaction term derived from cancer-to-reference allele frequency ratios. Each signal is weighted by cohort prevalence which are aggregated into a single ancestry-matched, reference-normalized score per patient. We applied PGViS to germline whole-genome sequencing from 1,102 TCGA and CPTAC patients, using the 1000 Genomes Project (n = 2,504 individuals) as the normal population reference. PGViS separated adenocarcinoma (AD) and squamous cell carcinoma from controls in European ancestry and East Asian AD. Genes at contributing loci were enriched for *PI3K-Akt, Wnt* , DNA damage response, and epithelial–mesenchymal transition programs. Smoking-stratified analysis concentrated this signal on canonical NSCLC driver pathways. PGViS is modular: it accommodates cohorts with broader ancestral representation and can adapt to other solid tumors, offering a cost-effective route to personal-genome risk assessment from germline variants alone.

## Full Text Availability

The license terms selected by the author(s) for this preprint version do not permit archiving in PMC. The full text is available from the preprint server.

